# Electric Fields Induced in the Brain by Transcranial Electric Stimulation: A Review of In Vivo Recordings

**DOI:** 10.3390/biomedicines10102333

**Published:** 2022-09-20

**Authors:** Matteo Guidetti, Mattia Arlotti, Tommaso Bocci, Anna Maria Bianchi, Marta Parazzini, Roberta Ferrucci, Alberto Priori

**Affiliations:** 1 Aldo Ravelli Research Center for Neurotechnology and Experimental Neurotherapeutics, Department of Health Sciences, University of Milan, Via Antonio di Rudinì 8, 20142 Milan, Italy; 2 Department of Electronics, Information and Bioengineering, Politecnico di Milano, Piazza Leonardo da Vinci 32, 20133 Milan, Italy; 3 Newronika S.p.A., 20093 Cologno Monzese, Italy; 4 III Neurology Clinic, ASST-Santi Paolo e Carlo University Hospital, 20142 Milan, Italy; 5 Istituto di Elettronica e di Ingegneria dell’Informazione e delle Telecomunicazioni (IEIIT), Consiglio Nazionale delle Ricerche (CNR), 20133 Milan, Italy

**Keywords:** neuromodulation, transcranial electric stimulation, electric fields, intracranial recordings, transcranial direct current stimulation, transcranial alternating current stimulation

## Abstract

Transcranial electrical stimulation (tES) techniques, such as direct current stimulation (tDCS) and transcranial alternating current stimulation (tACS), cause neurophysiological and behavioral modifications as responses to the electric field are induced in the brain. Estimations of such electric fields are based mainly on computational studies, and in vivo measurements have been used to expand the current knowledge. Here, we review the current tDCS- and tACS-induced electric fields estimations as they are recorded in humans and non-human primates using intracerebral electrodes. Direct currents and alternating currents were applied with heterogeneous protocols, and the recording procedures were characterized by a tentative methodology. However, for the clinical stimulation protocols, an injected current seems to reach the brain, even at deep structures. The stimulation parameters (e.g., intensity, frequency and phase), the electrodes’ positions and personal anatomy determine whether the intensities might be high enough to affect both neuronal and non-neuronal cell activity, also deep brain structures.

## 1. Introduction

Transcranial electrical stimulation (tES) is a neuromodulatory method that requires the non-invasive application of weak electrical currents through scalp electrodes [1,2]. Among all of the other types, transcranial direct current stimulation (tDCS) and transcranial alternating current stimulation (tACS) are the most studied techniques [3,4]. Since tDCS provides a direct current at a specific intensity, and tACS applies an alternating current at a specific frequency, they differ in the effects that they have on neural and non-neural cells [5,6]. Indeed, the temporal features (stimulus waveform) of the injected current, together with the spatial features (electrodes’ size, shape and montage) and individual head anatomy determine the electrical dose which induces biological and, ultimately, behavioral changes [7] (see [8,9] for systematic descriptions of tES effects). However, an accepted estimation of the electric field (E-field) that is developed in the cerebral tissues is still lacking [10]. Although it does not predict the stimulation effects per se [11], and such information would be crucial to: (I) fill the theorical gaps [12] and (II) deliver optimized stimulation protocols [12,13].

Computational simulations are currently the standard tool for spatial and temporal estimations of the tES-induced E-fields [14,15]. They have, for example, suggested the presence of maximal E-fields nearby the electrodes [16], or the role of cerebrospinal fluid and ventricular space in spreading the E-field to the deep structures [17]. Also, they have predicted intracerebral E-fields of no more than about 0.5 mV/mm for every 1 mA applied, but only in targeted regions [18,19,20,21], with weaker amplitudes being recorded across the brain [22]. However, computational simulations require a modelling process that includes remarkable caveats, such as in choosing the set of tissue conductivities [11,23]. Also, the predictions require invasive in vivo intracerebral measurements [10,24,25], which are highly susceptible to approximations [25,26] since the presence of electrodes may distort the current flow [27]. The closest estimations for humans come mainly from non-human primates (NHPs) [12,28,29], but still there is no clear knowledge about the E-fields that are realistically induced in the brain for the commonly used tES protocols [10]. Here, we review the available literature about intracerebral recordings of tDCS- and tACS-induced E-fields, discussing the results in the light of the current knowledge on electric field assessments and their effect on brain tissues (neuronal and non-neuronal).

## 2. In Vivo Recordings

**Figure 1:**
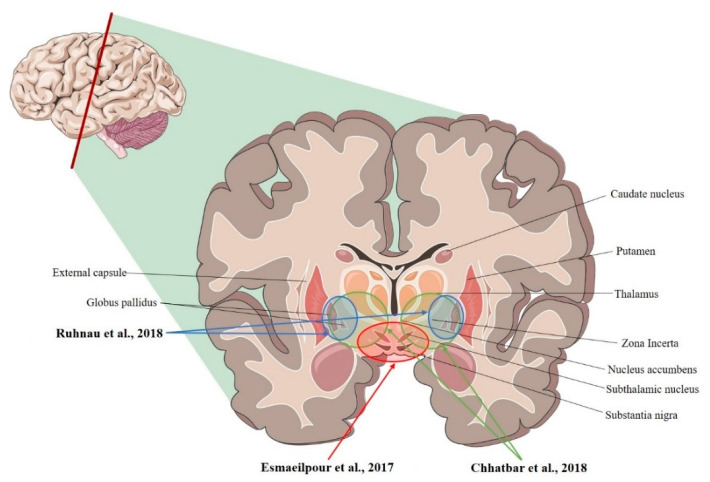
Coronal section of the brain showing the deep brain structures that were targeted for recording. Ruhnau et al., 2018 [36]; Chhatbar et al., 2018 [34]; Esmaeilpour et al., 2017 [37].

**Figure 2:**
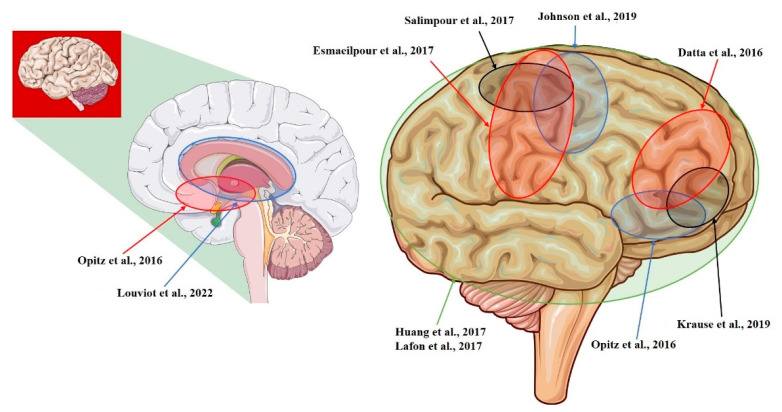
Sagittal section (left) and cerebral cortex (right) of the right hemisphere showing regions that were targeted for recordings. Datta et al., 2016 [14]; Opitz et al., 2016 [12]; Huang et al., 2017 [10]; Lafon et al., 2017 [32]; Krause et al., 2019 [29]; Louviot et al., 2022 [38]; Esmaeilpour et al., 2017 [37]; Salimpour et al., 2017 [26]; Johnson et al., 2019 [30].

**Figure 3:**
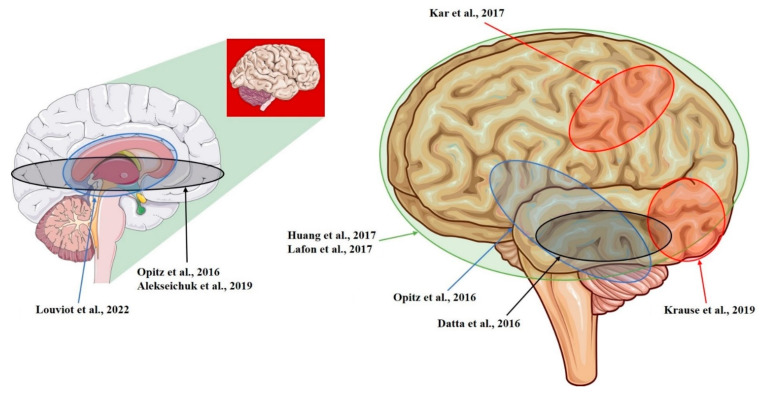
Sagittal section (**left**) and cerebral cortex (**right**) of the left hemisphere showing regions that were targeted for recording. Opitz et al., 2016 [12]; Kar et al., 2017 [28]; Krause et al., 2019 [29]; Datta et al., 2016 [14]; Huang et al., 2017 [10]; Louviot et al., 2022 [38]; Lafon et al., 2017 [32]; Alekseichuk et al., 2019 [39].

The current in vivo estimations of E-fields that are induced by tDCS and tACS in the human brain mainly come from recordings that have been conducted on animals (NHPs) and subjects undergoing neurosurgery (see Figure 1, Figure 2, Figure 3, Table 1, Table 2 and Table 3). Therefore, the available results need to be carefully considered because: (I) although NHPs are similar to humans, they still present remarkable differences [12,30] (see Section 5); (II) a diseased subject might present aberrant networks or an altered anatomy as a result of the conditions [24,31,32] or the treatments that they are undergoing [33]; (III) the presence of metallic implants and surgical procedures may interfere with the recordings [27] (see Section 5); (IV) heterogeneous stimulation protocols were applied (see Table 4); (V) tentative recording set-ups, that were typically designed for recording neural activity or local field potentials [34], were used (see Section 3). However, taken together, the results represent a first step towards an in vivo characterization of tDCS- and tACS-induced E-fields, potentially suggesting also a role of these techniques in the novel field of non-invasive deep brain stimulation (NDBS), with optimized protocols already being proposed [35].

**Table 1:** Studies investigating electric fields in deep brain structures.

Study	Model	Recording Area (tES Stimulation, Max E-Field Recorded)
Ruhnau et al., 2018 [36]	Human	R- and L-VIM nucleus; R- and L-GPi (tACS; ~0.08 mV/mm)
Chhatbar et al., 2018 [34]	Human	L-VIM nucleus; R- and L- STN, R- and L-GP (tDCS; 3.34 mV/mm)
Esmaeilpour et al., 2017 [37]	Human	R- and L- NAc; R- and L- STN; R-MC (tDCS; 5.06 mV/mm)

R = right; L = left; VIM = ventral intermediate; GPi = globus pallidus internus; tACS = transcranial alternating current stimulation; tDCS = transcranial direct current stimulation; STN = subthalamic nucleus; NAc = nucleus accumbens.

**Table 2:** Studies investigating electric fields in right hemisphere structures.

Study	Model	Recording Area (tES Stimulation, Max E-Field Recorded)
Datta et al., 2016 [14]	NHP	L-ITC; R-PFC (tDCS; 0.68 mV/mm)
Opitz et al., 2016 [12]	NHP + Human	R-lateral, L-medial orbitofrontal area; R- and L-superior, R- and L-inferior, and R- and L-middle temporal area; L-entorhinal area; L-cerebellum (tACS; 1.17 mV/mm)
Huang et al., 2017 [10]	Human	R- and L-, lateral and medial frontal, parietal, occipital, and temporal cortex; hippocampus * (tACS; 0.38 mV/mm)
Lafon et al., 2017 [32]	Human	R- and L-, lateral and medial frontal, parietal, occipital, and temporal cortex; hippocampus * (tACS; 0.16 mV/mm)
Krause et al., 2019 [29]	NHP	L-posterior ITC; R-ventrolateral PFC; lateral ventricle (tACS; 0.35 mV/mm)
Louviot et al., 2022 [38]	Human	R- and L amygdala, hippocampus, cingulate gyrus * (tACS; 0.49 mV/mm)
Esmaeilpour et al., 2017 [37]	Human	R- and L- NAc; R- and L- STN; R-MC (tDCS; 5.06 mV/mm)
Salimpour et al., 2017 [26]	Human	R-M1 and R-S1 (tDCS)
Johnson et al., 2019 [30]	NHP	R-M1; R-MC (tACS; median: 1.33 mV/mm)

NHP = non-human primate; L = left; R = right; ITC = inferotemporal cortex; PFC = prefrontal cortex; tDCS = transcranial direct current stimulation; tACS = transcranial alternating current stimulation; NAc = nucleus accumbens; STN = subthalamic nucleus; MC = motor cortex; M1 = primary motor cortex; S1 = primary sensory cortex; * = number and placement of recording electrodes were chosen solely by clinical requirements.

**Table 3:** Studies investigating electric fields in left hemisphere structures.

Study	Model	Recording Area (tES Stimulation, Max E-Field Recorded)
Opitz et al., 2016 [12]	NHP + Human	R-lateral, L-medial orbitofrontal area; R- and L-superior, R- and L-inferior, and R- and L-middle temporal area; L-entorhinal area; L-cerebellum (tACS; 1.17 mV/mm)
Kar et al., 2017 [28]	NHP	L-middle temporal area (tACS; 0.12 mV/mm)
Krause et al., 2019 [29]	NHP	L-posterior ITC; R-ventrolateral PFC; lateral ventricle (tACS; 0.35 mV/mm)
Datta et al., 2016 [14]	NHP	L-ITC; R-PFC (tDCS; 0.68 mV/mm)
Huang et al., 2017 [10]	Human	R- and L-, lateral and medial frontal, parietal, occipital, and temporal cortex; hippocampus * (tACS; 0.38 mV/mm)
Louviot et al., 2022 [38]	Human	R- and L amygdala, hippocampus, cingulate gyrus * (tACS; 0.49 mV/mm)
Lafon et al., 2017 [32]	Human	R- and L-, lateral and medial frontal, parietal, occipital, and temporal cortex; hippocampus * (tACS; 0.16 mV/mm)
Alekseichuk et al., 2019 [39]	NHP	L-occipital cortex, L-medial PFC; L-anterior hippocampus (tACS; 8.75 mV/mm)

NHP = non-human primate; R = right; L = left; tACS = transcranial alternating current stimulation; ITC = inferotemporal cortex; PFC = prefrontal cortex; tDCS = transcranial direct current stimulation; * = number and placement of recording electrodes were chosen solely by clinical requirements.

### 2.1. tDCS Recordings—Animal Studies

Despite animal models that have been extensively exploited to understand tDCS biophysics and physiology [22], only one study has reported in vivo recordings of a tDCS-induced E-field in NHPs [14]. Here, the authors tried to validate their computational models by implanting electrodes into two monkeys in the prefrontal cortex (PFC) and the inferotemporal cortex (ITC). The recordings reported an intensity of the induced E-field varying from 0.23 mV/mm to 0.68 mV/mm, for the montages that were assumed to create maximal intensity in the target regions (see Table 4). The results partially failed to confirm the predicted values, although the authors accounted for all of the head metallic implants while building the computational model. This is of particular interest because it confirms the difficulties of in silico estimations and in vivo recording reliability.

### 2.2. tDCS Recordings—Human Studies

Following the animal study, Esmaeilpour et al. [37] reported the first pioneer recordings from human beings undergoing tDCS. One mA of occipital-supraorbital stimulation lasting for around 30 s was found to induce a max E-field intensity of 0.11 mV/mm over the motor cortex, as recorded by epidural lead electrodes. The same protocol, with a doubled intensity of stimulation, induced a max E-field of 5.06 mV/mm in the nuclei accumbens (NAs), and one of 2.6 mV/mm did the same in the subthalamic nuclei (STNs) (see Table 4). Still, these results were affected by the poor recording methodology, similar to the study from Salimpour et al. in 2017, who surprisingly reported no significant changes in voltage during the motor DC stimulation (2 mA, 60 s) (see Table 4) [26]. In both cases, the electrodes were implanted without accounting for the direction of the induced E-field (see Section 5), therefore, without optimizing the recording.

More recently, however, another study gave strength to the idea that an E-field could significantly reach the deep brain structures (namely, the ventrointermediate nucleus—VIM nucleus, STNs, and the internal globi pallidi—GPis) during tDCS [34]. Three neurological patients (one with an essential tremor—ET, two with Parkinson’s disease—PD) with deep brain stimulation (DBS) implants were tested with different protocols of DC stimulation (see Table 4), showing that the E-field intensities were montage and dose-specific. A bitemporal stimulation generated the highest E-field, that doubled (from 0.11 to 0.19 mV/mm in STNs, and from 0.13 to 0.26 mV/mm in GPis) with a doubling intensity of the stimulation.

**Table 4:** Overview of the studies recording electric field during tACS and tDCS in humans and primates.

Study	Subjects	Stimulation Protocol	Electrodes Dimensions	Time of Stimulation		Recording Area	Induced Electric Field Intensity
Datta et al., 2016 [14]	Adult macaque (M)	tDCS, L-frontoparietal montage, 2 mA	Circular, 3.14 cm^2^	5 min (during fixation task)		L-ITC	Max predicted: 0.23 mV/mm (not confirmed)
		tDCS, R-frontooccipital montage, 2 mA				R-PFC	Max predicted: 0.68 mV/mm (confirmed)
	Adult macaque (F)	tDCS, L-frontoparietal montage, 2 mA				L-ITC	n.r.
		tDCS, R-frontoparietal, L-parietal montage, 2 mA				R-PFC	Max predicted: 0.42 mV/mm (confirmed)
Opitz et al., 2016 [12]	Cebus monkey (M)	tACS, L-occipitofrontal montage at 21 frequencies (1 to 10 Hz in 1 Hz steps, 10 Hz to 100 Hz in 10 Hz steps, plus 125 Hz and 150 Hz), 0.2 mA	Circular, 3.14 cm^2^		30 s each frequency	L-orbitofrontal cortex, frontal eye field and hippocampus	Max ± SE: 0.358 ± 0.001 mV/mm (median = 0.21 mV/mm)
	Cebus monkey (F)	tACS, L-occipitofrontal montage at 21 frequencies (1 to 10 Hz in 1 Hz steps, 10 Hz to 100 Hz in 10 Hz steps, plus 125 Hz and 150 Hz), 0.1 mA	Circular, 3.14 cm^2^		30 s each frequency	L-orbitofrontal cortex, frontal eye field, hippocampus, and thalamus	Max ± SE: 1.173 ± 0.003 mV/mm (median = 0.39 mV/mm)
	A single subject with medication-refractory epilepsy	tACS, bilateral frontoparietal montage, 1 Hz, 1 mA	25 cm^2^		2 min	L-medial and R-lateral orbitofrontal, L- and R- superior temporal, L-middle temporal, R-middle temporal, R- and L-inferior temporal cortical and subcortical regions, hippocampus, amygdala, cerebellum *	Max ± SE: 0.360 ± 0.008 mV/mm (median = 0.098 mV/mm)
	A single subject with medication-refractory epilepsy		25 cm^2^		2 min		Max ± SE: 0.163 ± 0.007 mV/mm (median = 0.059 mV/mm)
Esmaeilpour et al., 2017 [37]	A single subject	tDCS, R-occipital-supraorbital montage, 1 mA and 2 mA	Rectangular, 35 cm^2^	~30 s		NAc bilaterally	Max: 5.06 mV/mm
	A single subject	tDCS, R-occipital-supraorbital montage, 1 mA and 2 mA				STN bilaterally	Max: 2.6 mV/mm
	A single subject	tDCS, R-occipital-supraorbital montage, 1 mA				R-motor cortex	Max: 0.12 mV/mm
Salimpour et al., 2017 [26]	A single subject with PD undergoing surgery	tDCS, bilateral frontoparietal montage, 2 mA	Rectangular, 25 cm^2^	~1 min		R-primary motor cortex and primary sensory cortex	Unable to record
Kar et al., 2017 [28]	Adult macaque (M)	tACS, L-frontotemporal montage, 10 Hz, 2 mA	Square, 10.24 cm^2^	3 s		L-middle temporal area	Max: 0.12 mV/mm
		tACS, R-frontotemporal montage, 10 Hz, 2 mA	Square, 10.24 cm^2^	3 s			Max: 0.03 mV/mm
Huang et al., 2017 [10]	Nine subjects undergoing invasive monitoring for epilepsy surgery	tACS, frontooccipital montage, 1 to 10 Hz, 0.25 to 1 mA	Square, 4 cm2	n.r.		Lateral and medial frontal, parietal, occipital, and temporal cortex bilaterally; hippocampus *	Max (scaled at 1 mA): 0.28 mV/mm
		tACS, frontolateral-occipital montage, 1 to 10 Hz, 0.25 to 1 mA	Square, 4 cm^2^	n.r.			Max (scaled at 1 mA): 0.25 mV/mm
		tACS, frontolateral-occipital montage, 1 to 10 Hz, 0.25 to 1 mA	Square, 4 cm^2^	n.r.			Max (scaled at 1 mA): 0.10 mV/mm
	A single subject undergoing invasive monitoring for epilepsy surgery	tACS, L-frontoparietal, R-supraorbital, bilateral frontoparietal, and fronto-occipital montage, 1 to 10 Hz, 0.25 to 1 mA	Square, 4 cm^2^	n.r.			Max (scaled at 1 mA): 0.38 mV/mm
Lafon et al., 2017 [32]	Nine subjects with medication-refractory epilepsy	tACS, fronto-occipital montage, 0.75 to 1 Hz, 0.5 to 2.5 mA	Square, 4 cm^2^	Between 5 to 10 min		frontal, parietal, occipital, and temporal cortex bilaterally, deeper structures *	Median: 0.02 mV/mm (scaled to 1 mA of stimulation)
	A single subject with medication-refractory epilepsy	tACS, fronto-occipital montage plus three additional montages, 0.75 to 1 Hz, 0.5 to 2.5 mA (one patient)	Square, 4 cm^2^	Between 5 to 10 min			Median (scaled at 1 mA): 0.02 mV/mm Max intensity: 0.16 mV/mm at the highest current intensity (2.5 mA)
	Three subjects with medication-refractory epilepsy	tACS, frontolateral-occipital montage, 0.75 to 1 Hz, 0.5 to 2.5 mA (three patients)	Square, 4 cm^2^	Between 5 to 10 min			Median (scaled at 1 mA): 0.02 mV/mm
Ruhnau et al., 2018 [36]	A single subject suffering from movement disorders	tACS, bilateral temporal montage, 10 Hz, 1 mA	Rectangular, 35 cm^2^	n.r.		VIM nucleus and GPi, bilaterally	Max: ~0.08 mV/mm
Chhatbar et al., 2018 [34]	A single subject with ET	tDCS, bitemporal montage, 2 mA	Rectangular, 35 cm^2^	3 min		L-VIM nucleus	-
	A single subject with PD	tDCS, bitemporal montage, 2 mA				Bilateral STN	Max: −0.11 mV/mm
		tDCS, bitemporal montage, 4 mA					Max: −0.19 mV/mm
		tDCS, occipitofrontal montage, 2 mA					Max: −0.06 mV/mm
		tDCS, occipitofrontal montage 4 mA					Max: −0.02 mV/mm
	A single subject with PD	tDCS, bitemporal montage, 2 mA				Bilateral Gpi	Max: −0.13 mV/mm
		tDCS, bitemporal montage, 4 mA					Max: −0.26 mV/mm
		tDCS, occipitofrontal montage, 2 mA					Max: 0.04 mV/mm
		tDCS, occipitofrontal montage, 4 mA					Max: 0.03 mV/mm
Opitz et al., 2018 [24]	A single subject undergoing invasive monitoring for epilepsy surgery	tACS, bilateral frontoparietal montage, 1 Hz, 1 mA	Circular, 25 cm^2^	2 min		n.r. *	Mean: 0.058 mV/mm
	A single subject undergoing invasive monitoring for epilepsy surgery		Circular, 25 cm^2^	2 min			Mean: 0.115 mV/mm
Alekseichuk et al., 2019 [39]	Capuchin monkey (F)	Multielectrode tACS, 3 electrodes (L-fronto-occipito-temporal), 10 Hz, in 25 different phase conditions (from 0° to 360° in 15° steps) at 0.1 mA	Circular, 3.14 cm^2^	30 s each frequency		L-occipital cortex, medial PFC, and anterior hippocampus	Max: 6.03 mV/mm at 180° condition Min: 1.32 mV/mm at 0° condition
	Rhesus monkey (F)		Circular, 3.14 cm^2^	30 s each frequency			Max: 8.75 mV/mm at 180° condition Min: 3.03 mV/mm at 0° condition
Krause et al., 2019 [29]	Macaque monkey (M)	tACS, L-fronto–R-occipital montage, several frequencies at 2 mA	Circular, 3.14 cm^2^	5 min (during fixation task)		L-posterior ITC and R-ventrolateral PFC, lateral ventricle	Max: 0.28 mV/mm mean ± SE: 0.23 ± 0.01
	Macaque monkey (M)	tACS, L-frontoparietal-occipital montage, several frequencies at 2 mA	Circular, 3.14 cm^2^	5 min (during fixation task)			Max: 0.35 mV/mm mean ± SE: 0.19 ± 0.02 mV/mm
Johnson et al., 2019 [30]	Two monkeys (F)	tACS, bilateral frontotemporal montage, 10 Hz, 0.5 mA	Circular, 3.14 cm^2^	2 min		R-premotor and R-primary motor cortex	Median: 0.38 mV/mm (subject 1); Median: 0.43 mV/mm (subject 2)
		tACS, bilateral frontotemporal montage, 10 Hz, 1 mA	Circular, 3.14 cm^2^	2 min			Median intensity: 0.77 mV/mm (subject 1); Median intensity: 0.86 mV/mm (subject 2)
		tACS, bilateral frontotemporal montage, 10 Hz, 1.5 mA	Circular, 3.14 cm^2^	2 min			Median intensity: 1.15 mV/mm (subject 1); Median intensity: 1.33 mV/mm (subject 2)
Louviot et al., 2022 [38]	A single subject with medication-refractory focal epilepsy	tACS, bilateral temporal montage, 1 Hz, 3 Hz, 7 Hz, 35 Hz, 71 Hz, 140 Hz, 300 Hz, 0.5 and 1 mA; tACS, bilateral frontotemporal montage, 1 Hz, 3 Hz, 7 Hz, 35 Hz, 71 Hz, 140 Hz, 300 Hz, 0.5 and 1 mA	Circular, 4.52 cm^2^	2 min		Amygdala, hippocampus, cingulate gyrus *	Amygdala (1 mA): mean: 0.22 mV/mm; max: 0.25 mV/mm Hippocampus (1 mA): mean: 0.16 mV/mm; max: 0.26 mV/mm Cingulate gyrus (1 mA): mean: 0.06 mV/mm; max: 0.06 mV/mm
	Five subjects with medication-refractory focal epilepsy	tACS, bilateral temporal montage, 300 Hz, 0.5 and 1 mA; tACS, bilateral frontotemporal montage, 300 Hz, 0.5 and 1 mA	Circular, 4.52 cm^2^	2 min		Amygdala, hippocampus, cingulate gyrus *	Amygdala (1 mA): mean: 0.22 mV/mm; max: 0.29 mV/mm Hippocampus (1 mA): mean: 0.17 mV/mm; max: 0.38 mV/mm Cingulate gyrus (1 mA): mean: 0.08 mV/mm; max: 0.9 mV/mm
	A single subject with medication-refractory focal epilepsy	tACS, L-frontoparietal–R-temporal montage; bifronto-parietal montage; vertex–R-temporal montage; vertex–R-frontoparietal montage; fronto–R-temporal montage; fronto–L-temporal montage; fronto–R-frontoparietal montage; vertex–frontal montage; vertex–L-frontoparietal montage; bitemporal montage; L-temporo–R-frontoparietal, 300 Hz, 0.5 and 1 mA	Circular, 4.52 cm^2^	2 min		Amygdala, hippocampus, cingulate gyrus *	Amygdala (1 mA): mean: 0.18 mV/mm; max: 0.49 mV/mm Cingulate gyrus (1 mA): mean: 0.06 mV/mm; max: 0.11 mV/mm

M = male; F = female; tDCS = transcranial direct current stimulation; L = left; R = right; ITC = inferotemporal cortex; PFC = prefrontal cortex; S.E. = standard error; n.r. = not reported; tACS = transcranial alternating current stimulation; * = number and placement of recording electrodes were chosen solely by clinical requirements; NAc = nucleus accumbens; STN = subthalamic nucleus; GPi = globus pallidus internus; VIM = ventral intermediate; ET = essential tremor; PD = Parkinson’s disease.

### 2.3. tACS Recordings—Animal Studies

The first study on tACS was reported in 2016 when Opitz et al. [12] confirmed the role of the individual’s anatomy and the frequency of the stimulation by conducting two experiments on NHPs. Indeed, the presence of larger muscle mass overlaying the skull in one monkey (male) prevented the occurrence of high E-field intensities (max: 0.358 ± 0.001 mV/mm, median 0.21 mV/mm), and this was compared to that of the female monkey (max: 1.173 ± 0.003 mV/mm, median 0.39 mV/mm) at the medial PFC, frontal eye field and hippocampus. Also, AC stimulation was tested at more than 20 frequencies (see Table 4), with a reduction in the recorded intensities when the increasing frequencies exceeding 15 Hz—a phenomenon that is explained by frequency dependent increases in conductivity [40]. Lower E-field values, but those which are still potentially able to modulate neural activity [41,42], were recorded in a similar study from Kar et al. [28] (see Table 4). A montage-specific distribution of a tACS-induced E-field was confirmed; scalp electrodes over the recording area resulted in a fourfold increase in the E-field intensity (0.12 mV/mm) than that which was produced in the mirrored montage (0.03 mV/mm). A similar order of intensities was registered by Krause et al. [29] who assessed, cortically and subcortically, the effects in two monkeys (see Table 4). A peak field amplitude was recorded to be 0.28 mV/mm for monkey 1 (mean ± SE = 0.23 ± 0.01 mV/mm), and this was 0.35 mV/mm for monkey 2 (mean ± SE = 0.19 ± 0.02 mV/mm). Unlike the previous attempts, the in vivo values were consistent with the in silico predictions, which were determined considering the position and composition of all of the transcranial and intracranial implants. Thereby expanding the knowledge on how stimulation affects E-field intensities, a phase-dependency was found in two NHPs [39]; from 0° to 180°, the tACS E-field intensity increased (from 1.32 mV/mm to 6.03 mV/mm for monkey 1; whereas this was from 3.03 mV/mm to 8.75 mV/mm for monkey 2) and the E-field was progressively higher in the superficial brain regions; whereas, from 180° to 0°, the E-field changed direction and it was higher in the deeper brain regions (see Table 4). Also, the E-field direction was found to periodically switch from an anterior-to-posterior to a posterior-to-anterior orientation, with an E-field maximal value that was periodically and gradually moving from the most anterior to the most posterior contact, regardless of the montage. Finally, an E-field strength was found to linearly correlate with the AC intensity for a fixed montage [30] (see Table 4). The induced E-field over the motor cortex area transformed from a median of 0.38 mV/mm (0.5 mA) to a median of 1.15 mV/mm (1.5 mA) for monkey 1; whereas, this was from a median of 0.43 mV/mm (0.5 mA) to a median of 1.33 mV/mm (1.5 mA) for monkey 2.

### 2.4. tACS Recordings—Human Studies

While also implanting electrodes in NHPs, Opitz et al. [12] studied human subjects in order to compare the recordings. The sex-related differences that were found among the animals were not confirmed in the human subjects receiving 1 Hz AC of 1 mA for 2 min (see Table 4). The E-field maximal values (± SE) were 0.360 ± 0.008 mV/mm (median = 0.098 mV/mm) for patient 1, and 0.163 ± 0.007 mV/mm (median = 0.059 mV/mm) for patient 2, as were recorded near to the stimulating electrodes. The following year, Huang et al. [10] performed a recording study on 10 patients that were undergoing invasive monitoring for epilepsy surgery with subdural and depth electrodes. A tACS was performed following different protocols (see Table 4), with results suggesting that the deep brain areas may experience E-fields that are comparable in intensity to those of the cortical surface, as for the tDCS [34]. The fronto-occipital montages at 1 mA resulted in a max (± SD) E-field of about 0.28 ± 0.06 mV/mm; whereas, the frontolateral-occipital montage at 1 mA induced max (± SD) E-fields of 0.25 ± 0.10 mV/mm (cortex) and 0.21 ± 0.04 mV/mm (deeper structures—anterior cingulate, periventricular white matter). In reinforcing these findings, Ruhnau et al. [36] reported a voltage that was 10^4^–10^5^ times higher than the background activity in the deep brain structures (VIM nucleus and GPi) during tACS (1 mA, 10 Hz). However, in this case report, the movement disorder of the experimental subject and the duration of the stimulation were not reported (see Table 4). Lower values were reached by Lafon et al. [32] from 13 patients suffering from medication-refractory epilepsy (see Table 4), with a median E-field intensity across all electrodes of 0.02 mV/mm (scaled to correspond to 1 mA of stimulation) and a peak intensity of 0.16 mV/mm for 2.5 mA of stimulation. It is noteworthy that the individualized computational models predicted the occurrence of larger fields, as was also the case in another study by Opitz et al. [24]. Here, only the model accounting for both the implanted electrodes and skull defects led to verisimilar values. More recently, some authors tried to characterize the tACS-induced E-field besides only trying to quantify it inside the brain. A study from Louviot et al. [38] assessed the effect of stimulation frequency, intensity, and montage by targeting the drug-resistant epileptic patients’ hippocampus, amygdala and cingulate gyrus (see Table 4). As expected, the E-field intensity was correlated to the stimulation intensity, depending upon the montage, but not upon the stimulation frequency. For example, the strongest E-field in the deep brain structures were developed by those montages with the longest distance between the stimulating electrodes (probably due to a minimal scalp shunt).

## 3. Recording Set-Up

During tES, the E-field is not generated in a single uniform fashion, rather it varies across the brain [22], also, in the employment of tentative techniques to record it, artifacts may distort the signal [27] (see paragraph 5). Therefore, the characteristics of the recording set-up represent a fundamental aspect to be considered in approaching in vivo recording studies. The studies that are considered share a great heterogeneity in terms of instruments and procedures.

For the studies considering human subjects, the locations of the recording electrodes were chosen according to the clinical needs of the patient, both in case of DBS installment/replacement [26,34,36,37] or epilepsy neurosurgery [10,12,24,32,38]. Among the four studies exploiting DBS implants, three failed to report an exhaustive description of the acquisition methodology, instead describing the types of electrodes used—depth electrodes containing four recording points [36,37]. A more detailed report was provided by Chhatbar et al. [34] who used a data acquisition device with an input impedance of 1 MΩ, a common mode rejection of 80 dB, and a 14-bit resolution, and recorded a signal in the range of ±100 mV. Differently from the methods of other studies, the authors adopted no band-pass filtering to ensure that the characteristic DC pattern (i.e., flat waveform) was not filtered out. Among the studies considering epileptic patients, the authors used similar types of subdural electrodes (subdural grids, strips, and depth electrodes and s-EEG electrodes) [10,12,24,32]. Although three studies did not provide a detailed description of the recording set-up [12,24,26], Huang et al. [10] and Lafon et al. [32] reported a similar methodology. Both these studies used a bandpass filter of 0.16–250 Hz, with subdural electrodes sharing the same characteristics of the contacts—tens of the contacts were a few millimeters of diameter (2.3 mm, 2.4 mm or 1.1 mm) and of center-to-center spacing (5–10 mm). They referenced that intracranial EEG signals to a two-contact sub-galeal strip and a two-contact strip which was screwed to the skull were used for the instruments of the studies. Differently, Louviot et al. [38] amplified the signal that was recorded from a 256-channel s-EEG with a 10 kHz sampling rate, and applied a high-pass filter at 0.3 Hz, with the recording being set on the right foot.

For the studies considering NHPs, authors have used MRI-compatible head posts to facilitate the recording [12,14,28,29,30,39] and registered these from multi-contact stereo-EEG depth electrodes [12,39], microelectrode arrays [14,29], and single microelectrodes [28,30]. By positioning the stereo-EEG electrodes over the left occipital cortex (32 contacts for [39], 42 for [12]—mm spacing), Alekseichuk et al. [39] bandpass filtered the signal from 5 to 20 Hz, while Opitz et al. [12] used a data acquisition device with an input impedance of 10 MOhm, a common-mode rejection > 90 db, a high-pass frequency 0.016 Hz/10 s, and a low-pass frequency 250 Hz. On the other side, Datta et al. [14] recorded wideband signals from two arrays (unknown number of channels) simultaneously, sampling the signal at 30,000 Hz and band-pass filtering between 0.3 and 7500 Hz. Krause et al. [29] considered microelectrode arrays (two 64-channel arrays for one subject; two 96-channel arrays for the other subject) with electrodes that were placed in a square grid (0.4 mm spacing). The signal was bandpass filtered between 0.3–7500 Hz and sampled at 30,000 Hz. Differently, Kar et al. [28] used tungsten electrodes (0.2 mm shank diameter), while Johnson et al. [30] used a 96-channel microdrive that was used in the bandpass filtering of the signal in a narrowband around the applied stimulation frequency (10 Hz ± 1 Hz).

## 4. Electrical Stimulation Dose

In 1956, Terzuolo and Bullock demonstrated that neurons are significantly sensitive to weak E-fields (about 1 mV/mm) [43]. The effects of these polarizing currents critically depend on both the strength of the current applied and the duration of the application [44,45]. This means that even low currents that are applied for enough time exert significant biological effects. The “charge” (in Coulomb) summarizes this concept, with 1 Coulomb (C) being the amount of the electric charge that is transported in 1 s by a steady current of 1 Ampere (A) [45]. However, the role of the cellular and network entrainment has been emphasized [46,47,48], along with the parameters of the stimulation. Indeed, an induced E-field does not per se predict the stimulation effects [11,24], but it is a function of the electrical dose [49], which is given by a spatial distribution (defined by the shape, position, size and electrical properties of scalp electrodes) and temporal characteristics (waveform features) of the current that is injected [7]. In particular, tDCS and tACS differ only from the waveform features; tDCS-induced E-fields in the brain have a waveform that is similar to the stimulation current [7,12,50], while tACS induces a distorted E-field, due to the capacitive components of the current [51]. tES generates an E-field with specific temporal and spatial characteristics inside the brain. Therefore, not only the electrical dose, but also the individual’s anatomy strongly influences the effects of stimulation [7,52,53], as disclosed by a number of computational models [21,54,55,56,57,58,59]. For example, cortical folds affect the polarity of the stimulation [10], just as skull thickness and composition determines the amount of current reaching the brain [20,60], and the cerebrospinal fluid dissipates the current to the deep regions [60]. In summary, although the intensity of E-field that is reached in the brain represents an important parameter to understand the effects of the stimulation, the tES dose, along with the individual anatomy, determines the biological and neurophysiological changes [61,62] that occur at the neuronal and non-neuronal level [6].

## 5. Technical Issues and Limitations

Unlike other forms of invasive stimulation where the current delivery is tailored, the target of the stimulation is punctual and the neuronal response guides the interventions (e.g., deep brain stimulation—DBS, or direct electrical stimulation—DES) [63,64,65,66,67], the clinical meaning of the tES-induced E-fields values that are recorded in the brain is still unclear. For example, in intraoperative cortical and subcortical DES mapping in an awake patient, the targeted cortical and subcortical structures are directly stimulated while the patient performs a functional task (e.g., sensorimotor, cognitive and/or emotional) [68]. Local neural tissue activation or inhibition is reflected in the task performance, and this allows for the localization of eloquent areas of the brain [69,70]. Although the exact mechanism of this neurosurgical technique remains controversial, it represents the gold-standard to provide direct information on the functional organization of cortical and subcortical structures [71,72,73]. As for tES, ideally, in vivo models would be the most reliable to validate the computational simulations [25], and to assess the actual values of the E-fields in the brain. However, the intervening medium (brain tissues) between the source (stimulating electrodes) and the sensors (recording electrodes) is not uniform, and the variations can affect the spatial and temporal distribution [74]. Therefore, in practice, there are several factors that might mislead the recordings and their interpretation:

(I) *The anatomical characteristics of the subject*. Pathological subjects undergoing neurosurgery are often preferred for in vivo assessments for ethical reasons; however, their altered anatomy changes the E-field distribution, such as in stroke patients [75,76], patients with skull defects [24,77] or craniectomy [78]. Similar consequences can be seen in the use of cranial implants [77], such as electrodes [79] or bone screws [77], which alter the current flow in the surrounding tissues and lead to locally high current concentrations—a phenomenon that is known as the “edge effect” [80,81]. This effect occurs because the E-field masses around (i.e., at and near) the zone have a higher conductivity than bordering tissues which are less conductive. Replacing the removed skull with an insulating filler [82] or using a natural skull foramina as is the case for other neuromodulatory interventions [83] might minimizes these problems, but it is not clear whether natural openings promote an edge effect as well [84].
(II) *The translation from animal studies to clinical practice*. Besides the methodological differences between animal and human studies [85,86]—for example, animals typically undergo invasive stimulation techniques and have applied to them very strong intensities of stimulation [29] which are several-fold stronger than the humans undergo [87], the in vitro results do not account for the system-level properties [88], while the in vivo ones deal with a physiology and cytoarchitecture that may not be assimilable to the human brain [30,34]. Human neurons possess longer and compartmentalized apical dendrites, and their pyramidal neurons have larger dendritic arbores than rodents and primates do [89]. Also, their brain size, cortical folding, skin, skull, and CSF thicknesses are different [10,12,90]. For example, in a lissencephalic brain, the brain regions under the stimulating electrodes are exposed to an radially-inward (anodal) and radially-outward (cathodal) direct current flow, and the intermediate brain regions are exposed to a tangentially-direct current flow [91]. For the folded cortex, current crossing across the gyri can create a highly mixed pattern of directionality, even directly under electrodes [92,93].
(III) *The technical and methodological aspect of the recording*. The recording set-up challenges the observation of the voltage changes, being typically planned for recording neural activity or local field potentials [34]. For example, the use of microelectrode neural recording systems (single electrode or arrays) has shown their robustness and reliability to record neuronal activity in a number of studies, with multielectrode arrays able to target neuronal population per recording session [94]. However, microelectrodes can detect the electrical changes in the extracellular field [95], allowing for punctual recordings. During tES, there is no uniform induced E-field, but rather a range of intensities varying across the brain, with regions of maximum and regions of minimum values [16,92]. This is why any type of index that is considered (e.g., mean, median, maximum and minimum) may be misleading [22]. Also, the placement of the recording electrodes has been often not carefully planned [10,12,24,32]. This is of great concern because their position has a large effect on the measurements that are performed [25]. Intracranial electrodes, indeed, can measure potential differences only in the plane of the electrode strip [10], which should coincide with the general direction of the induced E-field to have maximal registering efficacy [34]. Also, the current density under the implanted electrode might not be equal to the average current density at the electrode, but instead it may be orders of magnitude higher at the electrode edges [96,97]. Similarly, other methods of recording that do not require electrodes might play a complementary and adjunctive role in investigating the neuromodulatory effects of tES in the deep brain areas thus potentially confirming the evidence that is here reported. For example, voltage-sensitive dye (VSD) imaging has been used to monitor cortical activity [98] and describe the cellular responses to invasive direct electrical stimulation [99], while intrinsic optical signal (IOS) imaging can be used to map the patterns of brain activity [100,101,102]. They reflect the functional response of the cells to the stimulation, rather than assess the E-field that is generated in the brain. Indeed, VSD imaging is based on dye molecules that are embedded in the cell membrane, which fluoresce proportionally to the changes in the transmembrane potential difference; IOS imaging refers to changes in the optical transmission, scattering, and reflectance of the tissue due to alterations in the blood volume [103], in the balance of oxy- and deoxyhemoglobins [100,101] and in ionic metabolism in astrocytes [104], among the others.
(IV) *The theoretical framework of E-fields assessment*. Current knowledge estimates that the minimum field strength for a direct neuromodulatory effect is likely somewhere between 0.10–1.00 mV/mm in the brain [87,105], with around 20 mV/mm for the plasticity effects [106,107]. Experimental [108] and clinical [109] tES protocols commonly provide a stimulation of <2 mA that produces E-fields on the order of 0.10–0.40 mV/mm [48,105], that are up to 1 mV/mm [4] in the brain. However, there is no consensus on the amount of stimulation that is needed to affect the human brain [110]. For example, a human cadaver study suggested that ab approximately 6 mA (i.e., three times the common amplitudes) stimulation would induce an effective intracerebral E-field [48], but the biophysical properties of brain tissue change profoundly after death [87], thus limiting a comparison [13].

## 6. Clinical Considerations

The current estimated values from the in vivo recordings are roughly at the lower end of what is required to affect neuronal activity in the animal experiments (<1 mV/mm), which is the case for the commonly used intensities of stimulation (1–2 mA).

Although most of the studies here that have been reviewed focused mainly on the quantification of the E-fields in the brain from a biophysical point of view (e.g., characterization of spatiotemporal properties, or validation of computational modeling estimations) [10,12,14,24,26,34,36,37,39], some authors have assessed also the neurophysiological effects of the stimulation [28,29,30,32]. tACS influenced the timing of neuronal spiking activity in a site-specific and frequency-specific manner [29], and dose-dependent fashion [30], suggesting that the induced E-fields directly affect neurons within the targeted area. Also, it reduced the visual motion adaptation on the amplitude and width of the tuning curves of single neurons, with the attenuation of the N2 component of the evoked LFP [28]. However, when testing the tACS effects on endogenous slow-wave rhythms during NREM sleep or theta activity during wakefulness, Lafon et al. [32] failed to demonstrate a role in the entrainment of spindle oscillations (during NREM sleep), nor in the modulation of theta, alpha, or gamma frequency activity with the phase of stimulation (during waking rest). All of these works encourage new studies to better understand the tACS effect, such as testing a wider range of frequencies and stimulation patterns to identify the most effective parameters for immediate and lasting physiological effects.

**Figure 4:**
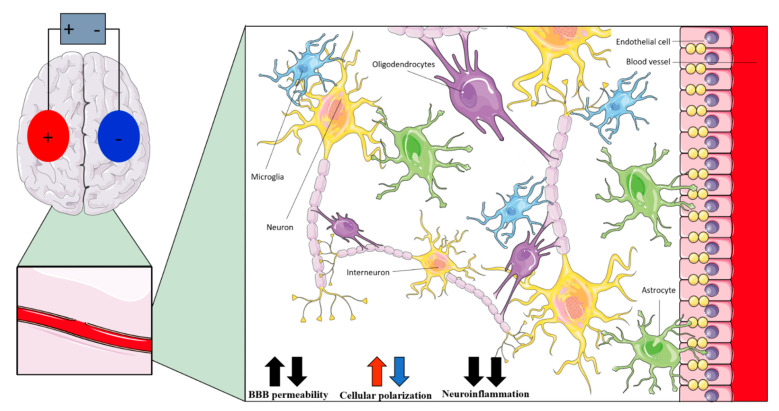
Representation (“+” is anode; “−“ is cathode) of the presumed in vivo effects of transcranial electrical stimulation on non-neural components of the brain at intensities applied in human protocols.

However, the clinical meaning of E-fields values that are recorded in the brain is still unclear. Besides the aspects that are described in paragraph 6 for different brain cell types (neurons, but also interneurons, glial and endothelial cells) that are sensitive to electrical stimulation [46,111,112,113] (see Figure 4 and Table 5), their role during tES is still open to questions being that their functions are intricately connected among them and with neurons [22]. Although the interneurons’ relatively symmetric morphology was predicted to result in a weaker somatic polarization [113], DCS modulates them in the hippocampal slices [114], while ACS affects fast-spiking interneurons via indirect network effects [115]. Glial cells are sensitive to DCS [116,117,118,119], because they have a transmembrane potential which changes by about 2 mV when the neurons are active [120] (up to 10 mV during seizures [121]). Theoretically, glial cells undergo a depolarization that is comparable to 2 mV during tDCS, as is suggested by a simplified cable theory study [121]. Therefore, during tDCS, the glial cells might be activated “as if” they were in physiological conditions [121], undergoing depolarization/hyperpolarization that might affect their activity for longer periods [121]. However, these results may not apply for tACS, because glial cells cannot generate results, while traveling depolarization and pulsating E-fields are very unlike to modulate glial transmembrane potential [121]. Typically, the glia is divided into three types with different morphologies and functions: astrocytes, microglia, and oligodendrocytes. DC stimulation might activate the ionic clearance processes in astrocytes [122] that regulate extracellular potassium and sodium concentrations. Also, tDCS might directly, and possibly indirectly, shift the microglia from their resting to their more active state [123,124], with amoeboid microglia more susceptible to this [6]. Microglia are immune cells in the CNS, with a fundamental and neuroinflammatory response [125]. Animal evidence suggests that tES modulates neuroinflammation, depending on the intensity of it and the pre-existing inflammatory condition [93], although the role of stimulation polarity is still unclear [86]. However, anodal tDCS reduced the expression of pro-inflammatory factors in aa rat model of vascular dementia [126], while a cathodal tDCS attenuated the activation of the astrocyte and microglia [127], downregulated the expression of the pro-inflammatory factors [127,128,129] and upregulated the expression of anti-inflammatory IL-10 [127] in rat models of stroke and of epilepsy [128]. The oligodendrocytes’ response to DCS is quite unknown [93], although ACS and anodal DCS might promote the proliferation and differentiation of oligodendrocyte-specific progenitors [130,131].

Therefore, the intensities of the E-fields that are reported by in vivo recordings should be generally strong enough to depolarize/hyperpolarize neurons, but also to affect non-neuronal cells’ activity. Glial cells might respond, thus undergoing functional changes, for example, downregulating neuroinflammation.

Another aspect that is still open to questions is the reaction of the endothelial cells forming the blood–brain barrier (BBB) [22]. Animal studies suggest that there is a positive modulatory effect of DCS on angiogenesis [132] and BBB permeability [133], thus inducing a polarity-specific flux across the endothelial cells that is proportional to the current density that is applied [134,135]. Curiously, an in vitro study found that the electrical force that is induced by the blood flow and acting on blood vessels strongly enhances NO signaling [136], according to the frequency and amplitude of it. This is of particular interest because this force has a sinusoidal form [136] and an intensity that is between 0.7–3 mV/mm [137] (i.e., similar to those induced by current human tES protocols) and, although no results are available, one could speculate that tACS would mimic such an effect.

**Table 5:** Presumed in-vivo effects of transcranial electrical stimulation on non-neural components of the brain at intensities applied in human protocols.

Non-Neuronal Cell	Anodal tDCS	Cathodal tDCS	tACS
Interneurons	Polarizing effects on dendrites and axons [22]		Modulation of fast-spiking interneurons activity [35]
Astrocytes	Polarizing effects (increased by network effect) [121]		-
Microglia	Shifting of microglia to active state [86,123]; modulation of neuroinflammation [126,127,128,129]		-
Oligodendrocytes	Promotion of neurogenesis [131]; promotion of oligodendrocyte-specific progenitors’ proliferation and differentiation [130]	Promotion of neurogenesis [131]	Promotion of oligodendrocyte-specific progenitors’ proliferation and differentiation [130]
Endothelial cells	Changes in cerebral blood perfusion [138]		-

## 7. Concluding Remarks

Determining the E-field that is realistically induced in the brain by tDCS and tACS is of particular concern, both for the ethical and clinical implications that it has. The current estimated values are roughly at the lower end of what is required to affect neuronal activity in the animal experiments (<1 mV/mm) for the commonly used intensities of stimulation (1–2 mA). However, some insights from the in vivo human and animal models are available in this paper. In this review, we have gathered these findings, focusing our attention on the human and non-human primate models. For clinical protocols, an injected current seems to reach the brain, even at the deep structures (e.g., NAs or STNs), following path of least resistance. Stimulation parameters, electrodes’ positions and personal anatomy determine the intensities. However, the induced E-fields should be generally strong enough to depolarize/hyperpolarize the neurons, but also to affect non-neuronal cells’ activity. Glial cells might respond “as if” they were in physiological conditions, and undergo functional changes, for example, by downregulating neuroinflammation. The indirect results suggest a similar situation for the endothelial cells, which might increase the blood–brain barrier permeability. Despite the heterogeneous stimulation protocols and rough recording methodology limits and the results of the in vivo recordings, these experiments might shed a light on the E-fields that are reached in the brain during tDCS and tACS.
